# Enhanced Hepatogenic Transdifferentiation of Human Adipose Tissue Mesenchymal Stem Cells by Gene Engineering with Oct4 and Sox2

**DOI:** 10.1371/journal.pone.0108874

**Published:** 2015-03-27

**Authors:** Sei-Myoung Han, Ye-Rin Coh, Jin-Ok Ahn, Goo Jang, Soo Young Yum, Sung-Keun Kang, Hee-Woo Lee, Hwa-Young Youn

**Affiliations:** 1 Department of Veterinary Internal Medicine, College of Veterinary Medicine, Seoul National University, Seoul, 151–742, Republic of Korea; 2 Department of Theriogenology and Biotechnology, College of Veterinary Medicine, Seoul National University, Seoul, 151–742, Republic of Korea; 3 Stem Cell Research Center, K-STEMCELL Co. Ltd., Seoul, 153–768, Republic of Korea; 4 Research Institute for Veterinary Science, College of Veterinary Medicine, Seoul National University, Seoul, 151–742, Republic of Korea; Instituto Butantan, BRAZIL

## Abstract

Adipose tissue mesenchymal stem cells (ATMSCs) represent an attractive tool for the establishment of a successful stem cell-based therapy in the field of liver regeneration medicine. ATMSCs overexpressing Oct4 and Sox2 (Oct4/Sox2-ATMSCs) showed enhanced proliferation and multipotency. Hence, we hypothesized that *Oct4* and *Sox2* can increase “transdifferentiation” of ATMSCs into cells of the hepatic lineage. In this study, we generated *Oct4*- and *Sox2*-overexpressing human ATMSCs by liposomal transfection. We confirmed the expression of mesenchymal stem cell surface markers without morphological alterations in both red-fluorescent protein (RFP) (control)- and Oct4/Sox2-ATMSCs by flow cytometry. After induction of differentiation into hepatocyte-like cells, the morphology of ATMSCs changed and they began to appear as round or polygonal epithelioid cells. Hepatic markers were evaluated by reverse transcription-polymerase chain reaction and confirmed by immunofluorescence. The results showed that albumin was strongly expressed in hepatogenic differentiated Oct4/Sox2-ATMSCs, whereas the expression level of α-fetoprotein was lower than that of RFP-ATMSCs. The functionality of hepatocytes was evaluated by periodic acid-Schiff (PAS) staining and urea assays. The number of PAS-positive cells was significantly higher and urea production was significantly higher in Oct4/Sox2-ATMSCs compared to that in RFP-ATMSCs. Taken together, the hepatocyte-like cells derived from Oct4/Sox2-ATMSCs were mature hepatocytes, possibly functional hepatocytes with enhanced capacity to store glycogen and produce urea. In this study, we demonstrated the enhanced transdifferentiation of *Oct4*- and *Sox2*-overexpressing ATMSCs into hepatocyte-like cells that have enhanced hepatocyte-specific functions. Therefore, we expect that Oct4/Sox2-ATMSCs may become a very useful source for hepatocyte regeneration or liver cell transplantation.

## INTRODUCTION

The liver is an important organ because it has a various physiological functions. It plays a central role in metabolic homeostasis, including glycogen storage, decomposition of red blood cells, plasma protein synthesis, hormone production, and detoxification [1–3]. For patients with end-stage liver disease, liver transplantation is the only available treatment; however, the use of this procedure is limited by the availability of donor organs and the high risk of transplant rejection [4,5]. For these reasons, an alternative therapeutic approach is needed for patients with chronic liver failure.

As an alternative therapy, the encouraging progress in stem cell research has paved the way towards the treatment of end-stage liver diseases and strategies using various types of stem cells have recently been utilized in animal models of chronic liver diseases [6–11]. In view of the pathogenic fundamentals of end-stage liver disease, stem cell-based treatment should aim to complement or replace damaged hepatocytes and to correct the imbalance between regeneration and degradation of extracellular matrix [12]. Recent work suggests that cells lost during aging or liver diseases can be reconstituted via stem cells resident within the specific organ and that tissue damage may attract migratory stem cell populations, particularly those from the bone marrow [7,13,14,15]. Therefore, it is believed that replacement of diseased hepatocytes and stimulation of endogenous or exogenous regeneration by human mesenchymal stem cells (MSCs) are promising candidates for liver-directed cell therapy [16].

MSCs can differentiate into cells of all mesodermal lineages, including adipocytes, osteocytes, chondrocytes, myocytes, and endothelial cells. In addition, adipose tissue mesenchymal stem cells (ATMSCs) have been shown to differentiate into other lineages such as neurons, hepatocytes and other epithelial cells, suggesting that MSCs are pluripotent adult stem cells [17–20] and show longer culture survival and higher proliferation capacity than other MSCs. Our group reported that ATMSCs genetically engineered to overexpress *Oct4* and *Sox2* (Oct4/Sox2-ATMSCs) demonstrated enhanced proliferation and multipotency [21]. Based on our previous study, we hypothesized that *Oct4* and *Sox2*, master regulators that control the self-renewal and pluripotency of stem cells [22], enhanced “transdifferentiation” of ATMSCs into hepatic lineages. The aim of this study was to investigate the ability of Oct4/Sox2-ATMSCs to undergo hepatic differentiation and to examine the functionality of hepatocyte-like cells derived from Oct4/Sox2-ATMSCs.

## MATERIALS AND METHODS

### Ethics Statement

Subcutaneous abdominal adipose tissue from donor was obtained with the patient's informed, written consent for research use under a protocol approved by the Institutional Review Board of the RNLBIO (IRB No. RNL-2012–06–001). The cells were provided to the investigators with all identifying information removed except cell passage number.

### Cell cultures

Human adipose tissue-derived mesenchymal stem cells were prepared from surplus of frozen, banked stem cells (K-STEMCELL, Seoul, Korea). In brief, human abdominal subcutaneous fat tissue was obtained by simple liposuction and isolated from the fat stromal vascular fraction by their adherence to plastic, and were cultured in RKCM medium (K-STEMCELL, Seoul, Korea). Expanded ATMSCs were aliquoted and stored in liquid nitrogen vapor phase until further use.

HepG2 cells, which are adherent epithelial-like cells derived from human hepatoblastoma, were obtained from the Korean Cell Line Bank (KCLB, Seoul, Korea). Cells were plated in minimum essential medium (HyClone, Logan, UT) supplemented with 10% fetal bovine serum (HyClone), 50 U/mL penicillin, and 50 μg/mL streptomycin (HyClone).

### Generation of Oct4/Sox2-overexpressing human ATMSCs

Oct4-IRES-Sox2 inserted in the PB-CA vector (originated from Addgene, http://www.addgene.org) was kindly provided by professor Goo Jang of Department of Theriogenology, College of Veterinary Medicine, Seoul National University. Then, human ATMSCs were transfected with the Oct4-IRES-Sox2-expressing vector using the transfection reagent D-fection (Lugen Sci, Seoul, Korea) according to the manufacturer’s instructions. The Oct4/Sox2-containing plasmid and transposae DNAs with the D-fection complex were added to the cells in an appropriate volume of ATMSCs culture medium. At 24 h after transfection, the medium was changed to fresh culture medium. Transfected cells were cryopreserved at passages 4 or 5. For cell analyses, we used transfected cells, which either were in culture (at passages 4 or 5) or were thawed from the cryopreserved stock. A PB-CA vector containing red-fluorescent protein (RFP) was used as control and generated by the same method.

### Flow cytometric analysis of cell surface marker expression in ATMSCs

Flow cytometry was used to analyze the expression of cell surface markers in RFP- and Oct4/Sox2-ATMSCs. By passage 5, a homogenous population of rapidly dividing cells with fibroblastoid morphology was obtained. ATMSCs were fixed with 70% ethanol at 4°C and stained for 30 min on ice with primary antibodies that recognized various surface molecules. Phycoerythrin-conjugated (PE) mouse anti-human CD29 (BD Bioscience, San Jose, CA), fluorescein isothiocyanate-conjugated (FITC) mouse anti-human CD31 (BD Bioscience), PE-conjugated mouse antihuman CD34 (BD Bioscience), FITC-conjugated mouse anti-human CD44 (BD Bioscience), FITC-conjugated mouse anti-human CD45 (BD Bioscience), PE-conjugated mouse anti-human CD73 (BD Biosciences Pharmingen, San Diego, CA), PE-conjugated mouse anti human CD90 (BD Biosciences), and PE-conjugated antihuman CD105/endoglin (R&D System, Minneapolis, MN) were used for the detection of cell surface antigens. The immunophenotype of MSCs was analyzed with the FACSCalibur flow cytometer (BD Biosciences, Bedford, MA) using the CELLQuest software (BD Biosciences).

### Reverse transcription-polymerase chain reaction analysis

Total RNA was isolated from RFP- or Oct4/Sox2-transfected ATMSCs using Trizol (Invitrogen, Carlsbad, CA) following the manufacturer’s protocol. Briefly, samples were transferred to a tube containing 1 mL RNA extraction solution. The homogenate was then extracted chloroform. Total RNA was precipitated with isopropanol. The obtained pellet was washed with ethanol, air-dried, and resuspended in 30 μL distilled water. RNA concentration and purity were determined on a nanophotometer (Implen, Munich, Germany) at 260 and 280 nm. Only samples with an absorbance ratio (260/280) of ≥ 1.8 were used for further analyses. First-strand cDNA was obtained by reverse transcription using 3 μg total RNA, M-MuLV reverse transcriptase, and an oligo (dT)-18 primer according to the manufacturer’s instructions (Invitrogen). Primer sequences are summarized in Table 1. Polymerase chain reaction (PCR) products were electrophoresed on 1.5% agarose gels to verify the size of the DNA fragments.

**Table 1:** Primer sequences for RT-PCR amplification of target genes.

Gene	Primer		Gene Bank Accession number	Product Size (bp)
**Oct4**	F	5’-TGCAGAAAGAACTCGAGCAA-3’	NM_002701.4	1082
	R	5’-ACACTCGGACCACATCCTTC-3’		
**Sox2**	F	5’-AGAACCCCAAGATGCACAAC-3’	NM_003106.3	466
	R	5’-ATGTAGGTCTGCGAGCTGGT-3’		
**AFP**	F	5’-GGCAGCCACAGCAGCCACTT-3’	NM_001134.2	276
	R	5’-TGCAGCGCTACACCCTGAGC-3’		
**ALB**	F	5’-CAACTATGTCCGTGAGCTTGGA-3’	NM_000477.5	339
	R	5’-GTGGTCGGTGCTGGTCTATATG-3’		
**transferrin**	F	5’-AGGGCCATTGCGGCAAACGA-3’	DQ923758.1	240
	R	5’-GTTCCACCCAGCGGAGGTGC-3’		
**GAPDH**	F	5’-AAGTGGATATTGTTGCCATC-3’	NM_002046.4	454
	R	5’-ACTGTGGTCATGAGTCCTTC-3’		

### Western blot analysis

After RFP- and Oct4/Sox2-ATMSCs were collected by centrifugation, the pellet was resuspended in lysis buffer (25 mM Tris, pH 7.5, 150 mM NaCl, 1% NP-40, 0.5% sodium deoxycholate, 0.1% sodium dodecyl sulfate) containing proteinase inhibitors and incubated on ice for 30 min. Following centrifugation at 16,000 × g for 15 min at 4°C, the supernatant containing the total cell extract was collected and kept at -80°C. Proteins from cell extracts were separated by gel electrophoresis and subsequently transferred to a Hybond polyvinylidene fluoride membrane (Amersham Biosciences, Piscataway, NJ). The membrane was incubated for 1 h at room temperature in blocking buffer (TBS-T containing 5% skim milk) to block nonspecific protein binding and then incubated at room temperature for 1 h with the primary antibody against Oct4 (Santa Cruz, Biotechnology Inc., Santa Cruz, CA) or Sox2 (Santa Cruz) diluted (1:300) in blocking buffer. Following four washes with TBS-T, the membrane was incubated for 1 h with the appropriate horseradish peroxidase-conjugated secondary antibody (diluted 1:3000) in blocking buffer. Antibody binding was visualized with the West-Q Chemiluminescent Substrate Plus Kit (GenDEPOT, Barker, TX).

### Hepatogenic differentiation

For differentiation assays, we used RFP- or Oct4/Sox2-ATMSCs at passage 4. Cells were seeded on collagen-coated plates (Invitrogen) and grown until confluence. Then, cells were serum-deprived for 48 h to prevent proliferation after which a two-step differentiation protocol was initiated that consisted of the sequential addition of growth factors, as described elsewhere [23–25]. Cells were cultured for two weeks in step-1 induction medium that contained Dulbecco’s modified Eagles medium supplemented with 2% fetal bovine serum, 4.9 mM nicotinamide (Sigma), 20 ng/mL hepatocyte growth factor (HGF, Sigma), and 10 ng/mL fibroblast growth factor 4 (Sigma). Then, cells were cultured for two weeks in step-2 maturation medium that consisted of Dulbecco’s modified Eagles medium supplemented with 2% fetal bovine serum, 30 ng/mL oncostatin M (Gibco), 10 nM dexamethasone (Sigma), and 10 μL/mL ITS + premix (Gibco, final concentration: 100 μM insulin, 6.25 μg/mL transferrin, 3.6 μM selenious acid, 1.25 mg/mL bovine serum albumin, and 190 μM linoleic acid) to generate mature cells. Media were changed twice weekly and morphological changes were monitored daily by observation under a microscope. At the end of the treatment, the expression of liver-associated markers was assessed by reverse transcription-PCR (RT-PCR) and functional tests for hepatocyte-like cells were carried out by period acid Schiff (PAS) staining and with the urea assay kit.

### Immunofluorescence

Cells were washed with PBS for three times and fixed with 4% paraformaldehyde, for 15min at room temperature. After washing with PBS, cells were permeabilized with 0.2% Triton-X 100 (Sigma), for 1h and blocked with 2% bovine serum for 1h at room temperature. The cells were incubated overnight sequentially at 4°C with primary antibodies raised against α-fetoprotein [(AFP) 1:50; goat, Santa Cruz] and albumin [(ALB) 1:50; mouse, Santa Cruz]. They were washed with PBS and incubated for 1 h at room temperature with secondary antibody (1:200, Santa Cruz). Subsequently, cells were stained for 15min with Hoecst33324 (Sigma) and observed under a fluorescence microscope.

### PAS staining

Adherent cells were fixed in 4% formaldehyde and oxidized in 0.5% periodic acid (Wako Pure Chemicals, Osaka, Japan) for 5 min, rinsed three times in dH_2_O, treated with Schiff’s reagent (Wako Pure Chemicals) for 15 min, rinsed in dH_2_O for 5–10 min, and assessed using a light microscope. For quantitative analysis, the percentage of PAS-positive cells was determined by counting 80–120 cells in five randomly selected fields.

### Urea assay

The amount of urea produced in the culture medium after treatment of the cells with 5 mM NH_4_Cl (Sigma) for 24 h was determined colorimetrically according to the manufacturer’s instructions (Quantichrom Urea assay kit, Bioassay Systems, Brussels, Belgium). Briefly, 50 μL of standard, culture supernatant, or medium was added to a test tube; then 200 μL of working reagent was added and the mixture was incubated for 20 min before the optical density was read at 415 nm. This assay is based on the reduction of ammonia produced via urea hydrolysis. Undifferentiated ATMSCs (20,000/cm^2^) and HepG2 cells (40,000/cm^2^) cultured for 24 h were treated with 5 mM NH_4_Cl and used as negative and positive controls, respectively.

### Statistical analysis

All experiments were performed at least twice. Values are expressed as average and standard deviations. The results were analyzed using Student t-test and one-way ANOVA with Tukey’s test in the GraphPad Prism v.5 software program (GraphPad Software, Inc., La Jolla, CA). For all studies, a value of *P* < 0.05 was accepted as statistically significant.

## RESULTS

### Analysis of the Oct4 and Sox2 expression in ATMSCs transfected with *Oct4/Sox2*

**Fig 1:**
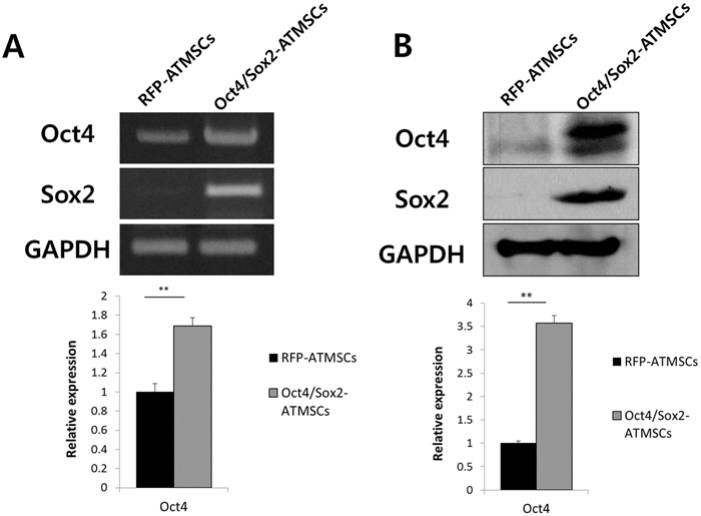
Analysis of the Oct4 and Sox2 expression in Oct4/Sox2-ATMSCs. (A) RT-PCR analysis revealed that the mRNA expression of Oct4 and Sox2 in Oct4/Sox2-ATMSCs were significantly higher than that in RFP-ATMSCs at 24 h post-transfection. (B) Western blot analysis showed high levels of Oct4 and Sox2 expression in Oct4/Sox2-ATMSCs. Data are representative of three independent experiments, with similar results. Statistical analysis was performed by student *t*-test (significant, **p < 0.01).

To assess the expression of Oct4 and Sox2 in ATMSCs transfected with Oct4/Sox2 (Oct4/Sox2-ATMSCs), we performed RT-PCR and western blot analyses (Fig. 1). The mRNA expression levels of Oct4 and Sox2 were significantly higher in Oct4/Sox2-ATMSCs than in RFP-ATMSCs; the expression of Oct4 and Sox2 in RFP-ATMSCs was almost undetectable. Concurrently, western blot analysis revealed that the expression of Oct4 and Sox2 was significantly upregulated in Oct4/Sox2-ATMSCs. These results showed that Oct4/Sox2-ATMSCs were successfully generated by liposomal transfection.

### Immunophenotyping of RFP- and Oct4/Sox2-ATMSCs

**Fig 2:**
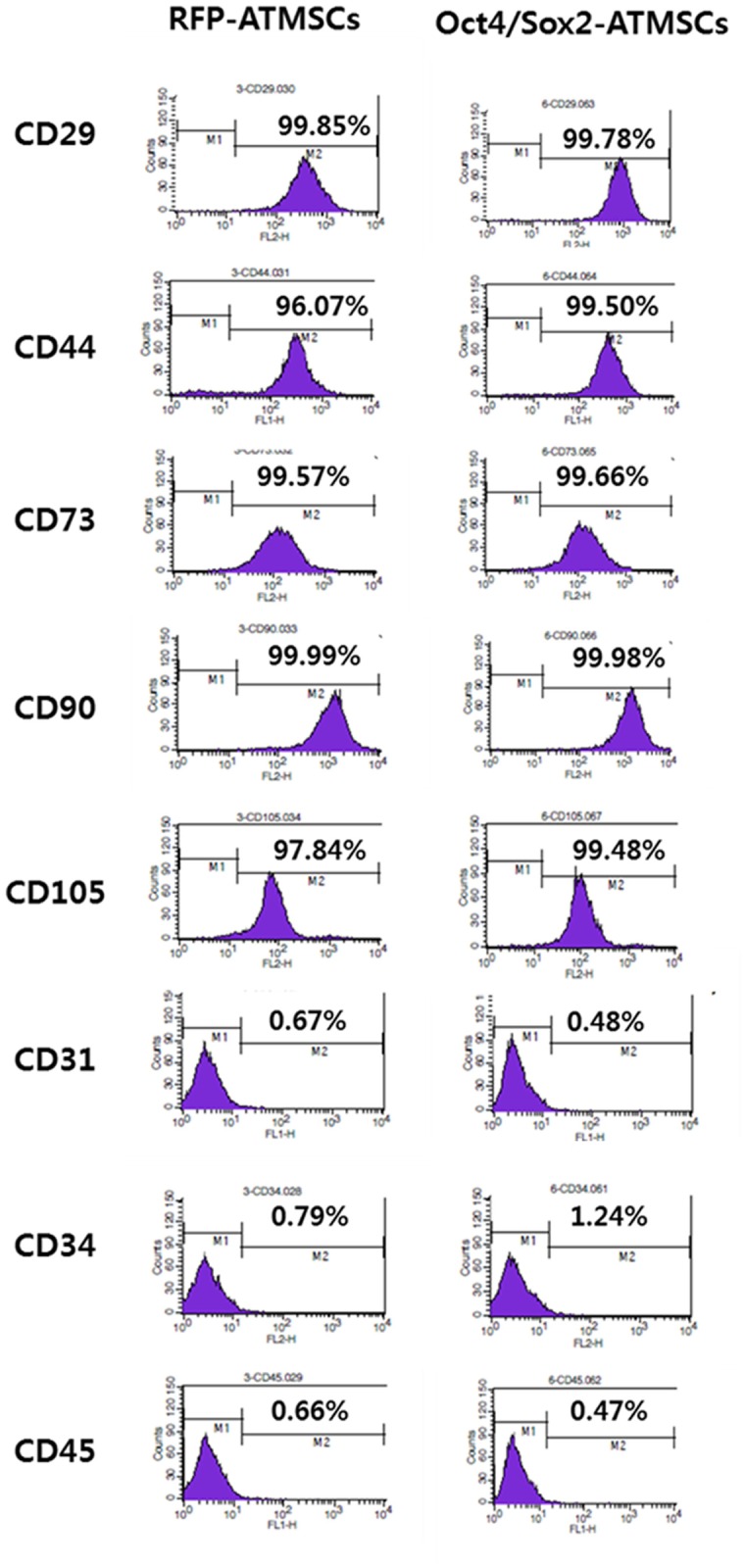
Immunophenotyping of RFP- and Oct4/Sox2-transfected ATMSCs. RFP-transfected ATMSCs and Oct4/Sox2-transfected ATMSCs at passage 5 were immunophenotyped for CD29, CD31, CD34, CD44, CD45, CD73, CD90, and CD105 by flow cytometry. The expression of ATMSC surface markers characteristic of MSCs was maintained.

The surface markers CD29, CD44, CD73, CD90, CD105, CD31, CD34, and CD45 were used to evaluate whether the immunophenotypic characteristics of ATMSCs changed after gene transfection at passage 5. Flow cytometry revealed high expression of CD29, CD44, CD73, CD90, and CD105, and the absence of the surface markers CD31, CD34, and CD45 in both of RFP- and Oct4/Sox2-ATMSCs (Fig. 2). The results of flow cytometric analyses indicate that the expression of ATMSC surface markers characteristic of MSCs was maintained.

### Hepatogenic differentiation of RFP- and Oct4/Sox2-ATMSCs

**Fig 3:**
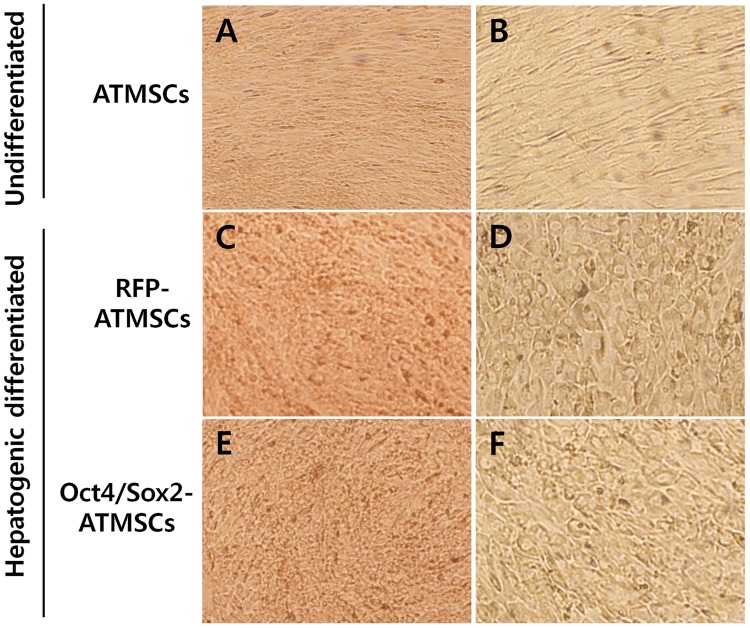
Morphology of RFP- and Oct4/Sox2-ATMSCs after 28 days hepatogenic differentiation. (A,B) Undifferentiated ATMSCs showed fibroblast-like morphology without morphological changes. (C,D) Hepatogenically differentiated RFP-ATMSCs and (E,F) hepatogenically differentiated Oct4/Sox2-ATMSCs exhibited significantly changed morphology and developed a round or polygonal epithelioid shape during step-2 of differentiation. Statistical analysis was performed by student *t*-test (significant, **p < 0.01).

ATMSCs were serum-deprived for two days and then cultured for 28 days in medium to which growth factors were added sequentially. Cell proliferation was inhibited by serum deprivation and exposure to culture conditions that induced hepatogenic differentiation resulted in gradual morphological changes, i.e., round or polygonal epithelioid cells were observed, during the culture period, whereas undifferentiated ATMSCs presented a fibroblast-like morphology (Fig. 3). After 28 days, more than 70% of the cells exhibited a polygonal shape.

**Fig 4:**
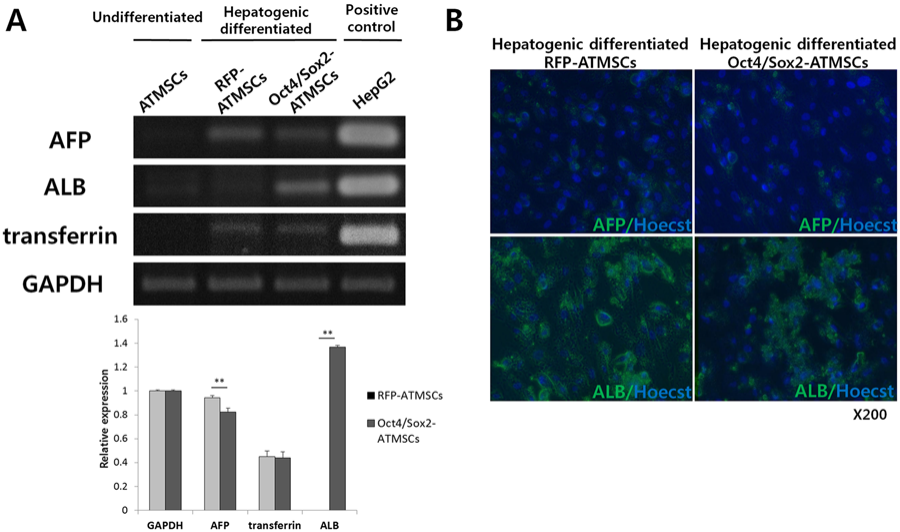
PCR analysis and immunofluorescence of liver markers after 28 days hepatogenic differentiation. (A) The mRNA expression level of albumin (ALB) was strongly expressed in hepatogenically differentiated Oct4/Sox2-ATMSCs, whereas the expression level of α-fetoprotein (AFP) was lower than that of RFP-ATMSCs. The expression levels of transferrin were not significantly different in both cells. Undifferentiated ATMSCs and HepG2 were used as negative and positive controls, respectively. (B) Hepatocyte-like cells from RFP- and Oct4/Sox2-ATMSCs are confirmed by immunofluorescence staining for AFP and ALB. Nuclei were counterstained with Hoecst33342.

To evaluate whether these morphological changes were associated with enhanced differentiation towards hepatocyte-like cells, RT-PCR analyses were carried out to investigate the expression of hepatic markers in hepatocyte-like cells derived from RFP- and Oct4/Sox2-ATMSCs (Fig. 4). Expression analysis of early (AFP) and late (ALB and transferrin) hepatic markers was performed and undifferentiated ATMSCs and HepG2 cells were used as negative and positive controls, respectively. The early hepatocyte differentiation marker AFP was found in both hepatogenically differentiated RFP- and Oct4/Sox2-ATMSCs. In hepatocyte-like cells derived from RFP-ATMSCs, the expression level of AFP was higher than that of Oct4/Sox2-ATMSCs; however, they did not express ALB, a marker of well-differentiated hepatocytes. In contrast, the expression of ALB was upregulated in hepatogenically differentiated Oct4/Sox2-ATMSCs. The expression levels of transferrin in both types of cells were not significantly different.

To confirm the expression of key genes, immunocytochemistry was performed for proteins expression in hepatocyte-like cells from RFP- and Oct4/Sox2 ATMSCs at day 28 differentiation. As shown in Fig. 4B, hepatic markers positive polygonal cells can be observed in both differentiated ATMSCs.

Together with the results from the expression analysis of hepatic markers, these data demonstrate that more Oct4/Sox2-ATMSCs reached a mature state, whereas RFP-ATMSCs remained in an immature or transient state.

### Functionality test of hepatocyte-like cells derived from RFP- and Oct4/Sox2-ATMSCs

**Fig 5:**
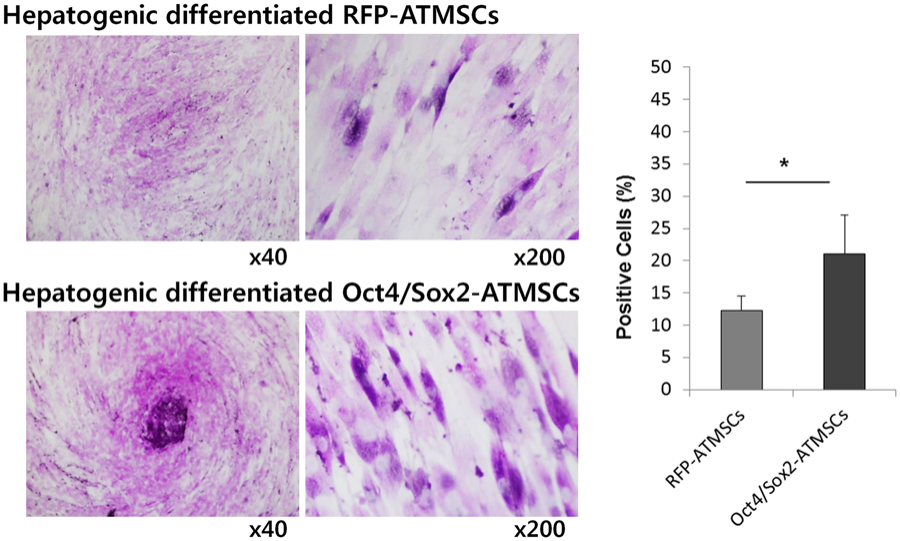
Period acid Schiff (PAS) staining of RFP- and Oct4/Sox2-ATMSCs after 28 days hepatogenic differentiation. (A) Detection of glycogen in the cytoplasm of MSCs subjected to the liver differentiation protocol was demonstrated by PAS staining. PAS-positive substances stain pink in the cytoplasm of cells. (B) The number of PAS-positive cells is expressed as percentage of the total number of counted cells and was significantly higher in Oct4/Sox2-ATMSCs than that of RFP-ATMSCs. The experiments were repeated at least three times and similar findings were observed. Data represent the mean ± SD of three independent experiments. Statistical analysis was performed by student *t*-test (significant, *p < 0.05).

To evaluate the functionality of hepatocytes, we performed PAS staining of hepatocyte-like cells derived from RFP- and Oct4/Sox2-ATMSCs at 28 days to assess their ability of glycogen storage (Fig. 5). The number of PAS-positive cells is expressed as percentage of the total number of counted cells and was significantly higher in Oct4/Sox2-ATMSCs than in RFP-ATMSCs (1.7-fold).

**Fig 6:**
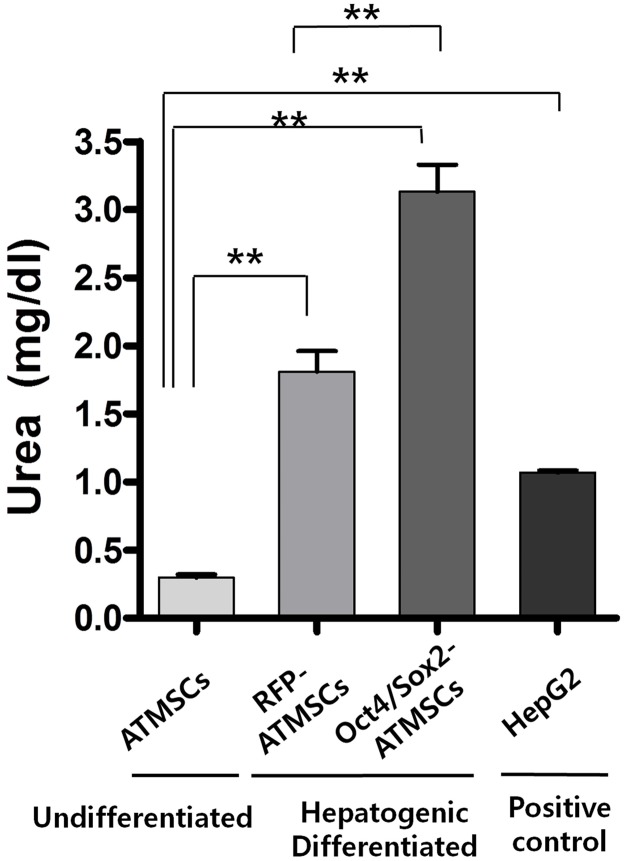
Production of urea in hepatocyte-like cells derived from RFP- and Oct4/Sox2-ATMSCs after 28 days hepatogenic differentiation. After hepatogenic induction, urea production was significantly increased in cultures of differentiated RFP- and Oct4/Sox2-ATMSCs. Thus, the amount of urea produced by hepatocyte-like cells derived from Oct4/Sox2-ATMSCs was significantly higher than that of RFP-ATMSCs. Undifferentiated ATMSC and HepG2 Cells were used as negative and positive control, respectively. The experiments were repeated at least three times and similar findings were observed. Data represent the mean ± SD of three independent experiments. Statistical analysis was performed by ANOVA followed by Tukey’s post hoc test of the means (significant, **p < 0.01).

We also determined whether differentiated hepatocyte-like cells derived from both types of ATMSCs produced and secreted urea at 28 days (Fig. 6). The level of urea produced by undifferentiated ATMSCs, which served as negative control, was almost undetectable. After induction of hepatogenic differentiation, urea production was significantly increased in cultures of differentiated RFP- and Oct4/Sox2-ATMSCs and reached the level of hepatocytes under the same culture conditions. Thus, the amount of urea produced by hepatocyte-like cells derived from Oct4/Sox2-ATMSCs was significantly higher than that produced by RFP-ATMSCs. These results indicate that hepatocyte-like cells derived from Oct4/Sox2-ATMSCs have an enhanced capacity to store glycogen and to produce urea, which are important requirements of functional hepatocytes.

## DISCUSSION

Recent and rapid developments in stem cell biology have made it possible to use stem cells in the treatment of several types of diseases [26–29]. In this context, adult stem cells have been demonstrated to have the capacity to regenerate damaged tissues or organs, including the liver. Many reports have suggested that ATMSCs are multipotent and can differentiate into multiple mesenchymal lineages, including hepatocytes, after transplantation in patients with chronic liver disease [23,24,30,31]. However, although MSCs have been shown to retain pluripotency and their efficacy has been demonstrated in clinical trials, mixed results such as only transient effects due to poor cellular retention or have been obtained that indicate the need for further optimization [32]. For these reasons, investigations to increase the number of pluripotent MSCs and the efficacy of stem cell-based therapies for the treatment of liver diseases are required.

In 2010, Meyerrose *et al*. [33] suggested that genetically manipulated MSCs could serve as cellular therapeutics. In addition, recent studies have demonstrated that the application of transcription and growth factors such as Foxa2, insulin-like growth factor-1, and HGF contributed to the recovery of damaged liver tissues, because they can induce hepatoprotective factors and transcriptional activators of liver-specific genes that are involved primarily in hepatic metabolism, as well as early developmental events [34–36]. In our previous study, Oct4/Sox2-overexpressing ATMSCs showed improved cell proliferation and greater potential to differentiate into adipocytes or osteoblasts [21]. In particular, *Oct4* and *Sox2* are well known key transcription factors essential for self-renewal and survival of stem cells. Oct4 has been demonstrated to be crucial for the maintenance of stemness of pluripotent stem cells [37] and plays a unique role in the development and determination of pluripotency. Sox2 also plays an important role in the maintenance of pluripotency of human stem cells [38]. Furthermore, Oct4 and Sox2 have been described previously to drive the pluripotent-specific expression of a number of genes through cooperative interaction [39]. For these reasons, we used Oct4 and Sox2 to improve the transdifferentiation strategy of MSCs for hepatogenesis.

In this study, we successfully engineered Oct4- and Sox2-overexpressing ATMSCs by liposomal transfection. To introduce exogenous DNA into cells efficiently, many researchers examined several approaches [40,41]. Viral transduction, in particular adenovirus-mediated gene transfer, can generate stable cell clones with high efficiency and low cell mortality, making it a popular option in gene therapy [42]. However, safety concerns associated with viral transduction have prompted us to look for alternative non-viral gene delivery approaches. Recommendable traditional transfection methods are calcium phosphate precipitation, liposomal transfection, and electroporation. Some researchers are concerned because these techniques usually result in less than 1% transfection efficiency and high cell mortality [40]. However, when we used the liposomal transfection method with the PB-CA vector, which is a high-copy-number vector, RT-PCR demonstrated high expression of exogenous *Oct4* and *Sox2*, and high Oct4 and Sox2 protein levels were detected by western blot. In addition, advantages of the non-viral transfection methods used in this study include simplicity of use, ease of large-scale production, and absence of a vector-mediated immune response.

According to the two-step protocol for hepatogenic differentiation, hepatocyte-like cells were obtained from Oct4/Sox2-ATMSCS by sequential addition of a cytokines-based cocktail to the medium. In step-1 induction medium for the early stage of differentiation, fibroblast growth factor, HGF, and nicotinamide were used as main induction factors. In fact, fibroblast growth factors play a critical role in the morphogenetic growth of the hepatic endoderm [43,44]. HGF is a crucial factor for hepatogenesis by stimulating rapid hepatoblast proliferation. Nicotinamide, after dissolving in water, play roles in maintaining energy for cellular functions, including DNA repair and genomic stability. For hepatogenic differentiation, nicotinamide has been shown to enhance the proliferation of primary rat hepatocytes and formation of small hepatocyte colonies [45]. In step-2 induction medium for the late stage of differentiation, we used oncostatin M, dexamethasone, and ITS+ premix as major induction factors. Oncostatin M is secreted by hematopoietic cells and involved in the maturation fate of fetal hepatocytes [44]. Dexamethasone is a synthetic glucocorticoid hormone that induces enzymes involved in gluconeogenesis in the liver [18,46]. The ITS+ premix seems to be required for the survival of cells grown as monolayer during the induction period.

To determine whether our cell culture protocols induce the transcription of factors that are essential for hepatocyte differentiation, we investigated the presence of mRNA of hepatocyte-specific genes, i.e., AFP, ALB, and transferrin. According to Schmelzer *et al*., [47] the gene expression profile of hepatic cells ranges from that of the stem cell stage to that of the mature functional stage. They demonstrated that hepatoblasts expressed low levels of CK19, elevated levels of ALB, high levels of AFP, and low levels of adult liver-specific proteins. Mature hepatocytes did not express AFP and CK19 and had acquired the well-known adult-specific expression profile that includes high levels of ALB, CYP3A4, connexins, phosphoenolpyruvate carboxykinase, and transferrin. The expression of hepatic markers (ALB and transferrin) reflects the nature of hepatic stem cells toward hepatocyte lineages, i.e., hepatocytes. Hepatoblasts proved to be cells at an intermediate state of differentiation at which adult liver-specific genes are activated but expressed at low levels and with the unique, defining feature of AFP. The gene expression profiles of hepatic markers in hepatocyte-like cells derived from differentiated RFP- and Oct4/Sox2-ATMSCs showed distinct results. The expression of AFP in hepatocyte-like cells derived from RFP-ATMSCs was higher than that in Oct4/Sox2-ATMSCs; however, RFP-ATMSCs did not express ALB, a marker of well-differentiated hepatocytes. In contrast, the expression of ALB was upregulated in hepatogenically differentiated Oct4/Sox2-ATMSCs at 28 days post-induction. The expression levels of transferrin in both types of cells were not significantly different. These results indicate that, although both cell types differentiated into hepatocyte-like cells, cells derived from Oct4/Sox2-ATMSCs were mature hepatocytes that might represent functional hepatocytes, whereas cells derived from RFP-ATMSCs remained hepatoblasts or incompletely differentiated hepatocytes.

The obtained cells exhibited specific hepatic functions, including glycogen storage and the production of urea, whereas undifferentiated ATMSCs did not express hepatocyte markers and had almost no functional hepatocyte activity. PAS staining revealed that the number of PAS-positive cells among hepatocyte-like cells derived from Oct4/Sox2-ATMSCs was higher than that of PAS-positive cells derived from RFP-ATMSCs, which was confirmed by counting cells in randomly selected field. In addition, we determined the amount of urea secreted by differentiated cells and found that it was similar or greater than that secreted by HepG2 cells. Both results indicate that hepatocyte-like cells derived from RFP-ATMSCs were still not functionally matured compared to those derived from Oct4/Sox2-ATMSCs. Taken together, these functional tests demonstrated that hepatocyte-like cells derived from Oct4/Sox2-ATMSCs have functional characteristics that are consistent with the results for the hepatocyte markers.

Here, we demonstrated enhanced transdifferentiation of Oct4- and Sox2-overexpressing ATMSCs into hepatocyte-like cells with enhanced hepatocyte-specific functions. These results suggested that ATMSCs genetically engineered to overexpress *Oct4* and *Sox2* can be used to replace damaged hepatocytes or to secrete hepatoprotective cytokines in end-stage liver disease. Although additional studies are required using animal models to evaluate our results, the possibility of the induction of specific differentiation into hepatocyte-like cells using *Oct4* and *Sox2* gene engineering may make ATMSCs an ideal cell choice for *in vivo* therapies for genetic or acquired disorders of the liver. Altogether, these findings provide a new and useful alternative source that can enable hepatocyte regeneration or liver cell transplantation with which the limitation of liver cell donors for future use in clinical applications may be overcome.
