# Correction: Enhanced Hepatogenic Transdifferentiation of Human Adipose Tissue Mesenchymal Stem Cells by Gene Engineering with Oct4 and Sox2

**DOI:** 10.1371/journal.pone.0183734

**Published:** 2017-08-17

**Authors:** Sei-Myoung Han, Ye-Rin Coh, Jin-Ok Ahn, Goo Jang, Soo Young Yum, Sung-Keun Kang, Hee-Woo Lee, Hwa-Young Youn

In the graph legend in Panel A of Fig 4, the color for RFP-ATMSCs is incorrect. Please see the corrected Fig 4 here.

**Fig 4:**
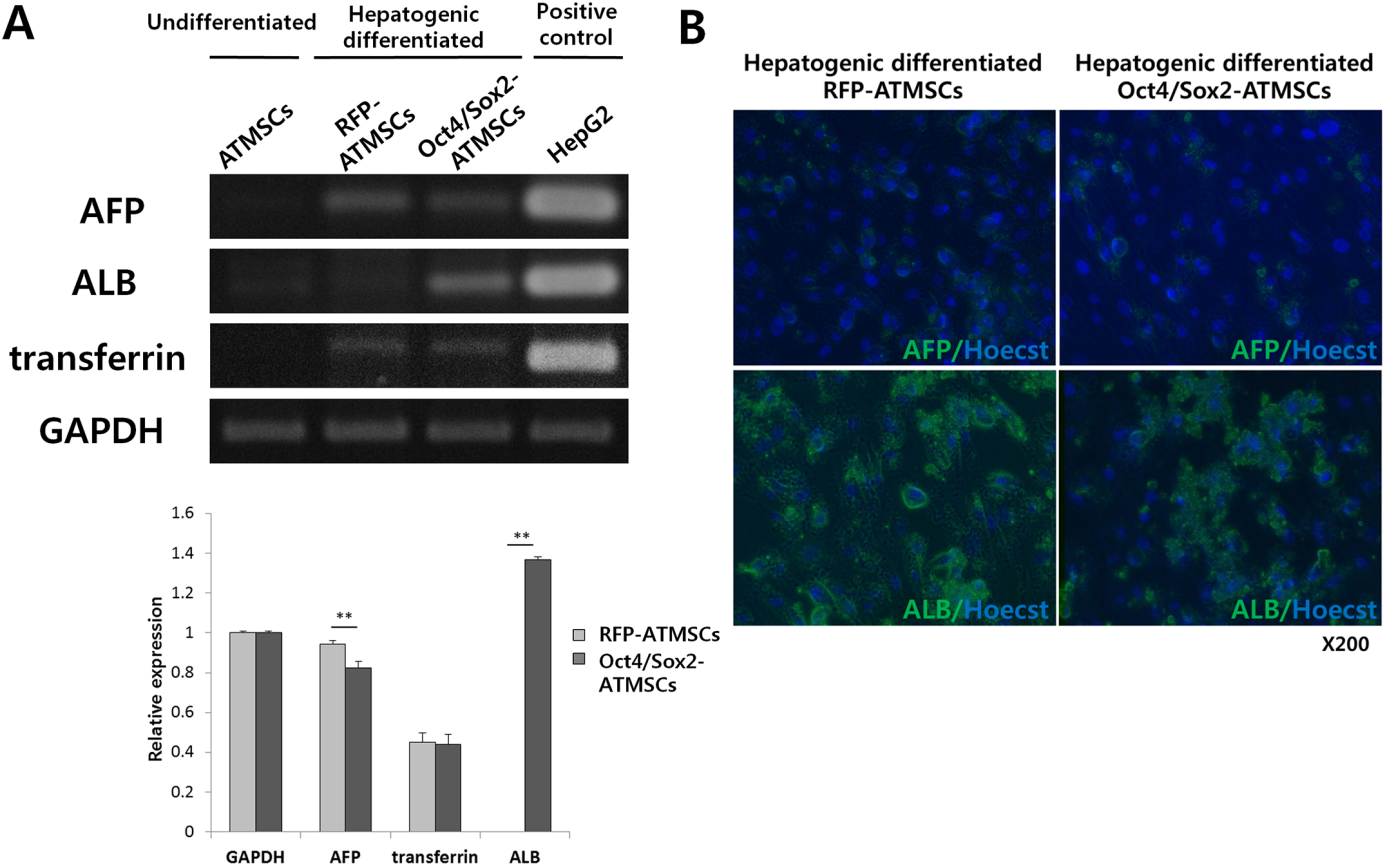
PCR analysis and immunofluorescence of liver markers after 28 days hepatogenic differentiation. (A) The mRNA expression level of albumin (ALB) was strongly expressed in hepatogenically differentiated Oct4/Sox2-ATMSCs, whereas the expression level of α-fetoprotein (AFP) was lower than that of RFP-ATMSCs. The expression levels of transferrin were not significantly different in both cells. Undifferentiated ATMSCs and HepG2 were used as negative and positive controls, respectively. (B) Hepatocyte-like cells from RFP- and Oct4/Sox2-ATMSCs are confirmed by immunofluorescence staining for AFP and ALB. Nuclei were counterstained with Hoecst33342.

## References

[1] Han S-M, Coh Y-R, Ahn J-O, Jang G, Yum SY, Kang S-K, et al (2015) Enhanced Hepatogenic Transdifferentiation of Human Adipose Tissue Mesenchymal Stem Cells by Gene Engineering with Oct4 and Sox2. PLoS ONE 10(3): e0108874 https://doi.org/10.1371/journal.pone.0108874 25815812 10.1371/journal.pone.0108874 PMC4376765

